# Application of the Nucleation Theorem to Crystallization of Liquids: Some General Theoretical Results

**DOI:** 10.3390/e21121147

**Published:** 2019-11-25

**Authors:** Jürn W. P. Schmelzer

**Affiliations:** Institute of Physics, University of Rostock, Albert-Einstein-Strasse 23-25, 18059 Rostock, Germany; juern-w.schmelzer@uni-rostock.de; Tel.: +49-381-498-6889; Fax: +49-381-498-6882

**Keywords:** crystal nucleation, crystal growth, 64.60.Bd General theory of phase transitions, 64.60.Q Nucleation, 81.10.Aj Theory and models of crystal growth, 82.60.Nh Thermodynamics of nucleation in physical chemistry and chemical physics

## Abstract

Different aspects in applying the nucleation theorem to the description of crystallization of liquids are analyzed. It is shown that, by employing the classical Gibbs’ approach in the thermodynamic description of heterogeneous systems, a general form of the nucleation theorem can be formulated that is valid not only for one-component but generally for multi-component systems. In this analysis, one basic assumption of classical nucleation theory is utilized. In addition, commonly employed in application to crystallization, it is supposed that the bulk properties of the critical clusters are widely identical to the properties of the newly evolving crystal phase. It is shown that the formulation of the nucleation theorem as proposed by Kashchiev [J. Chem. Phys. **76**, 5098-5102 (1982)], also relying widely on the standard classical approach in the description of crystal nucleation, holds for multi-component systems as well. The general form of the nucleation theorem derived by us is taken then as the starting point for the derivation of particular forms of this theorem for the cases that the deviation from equilibrium is caused by variations of either composition of the liquid phase, temperature, or pressure. In this procedure, expressions recently developed by us for the curvature dependence of the surface tension, respectively, its dependence on pressure and/or temperature are employed. The basic assumption of classical nucleation theory mentioned above is, however, in general, not true. The bulk and surface properties of the critical crystal clusters may differ considerably from the properties of the evolving macroscopic phases. Such effects can be incorporated into the theoretical description by the application of the generalized Gibbs approach for the specification of the dependence of the properties of critical crystal clusters on the degree of metastability of the liquid phase. Applying this method, it is demonstrated that a similar formulation of the nucleation theorem, as derived based on classical nucleation theory, holds true also in cases when a dependence of the state parameters of the critical clusters on the degree of deviation from equilibrium is appropriately accounted for.

## 1. Introduction

Crystallization processes play a decisive role in a broad spectrum of phenomena occurring in nature and technology. They are governed by nucleation and growth of crystallites evolving in the ambient liquid phase. Due to its importance, intensive research is devoted to the analysis of crystallization (see, e.g., [1,2,3,4]) attempting to exhibit the major factors either catalyzing or inhibiting crystal nucleation and growth.

The interpretation of experimental data on crystal nucleation and growth processes is performed up to now predominantly via the classical nucleation theory (CNT) [5,6,7,8,9]. It has been developed based on the work of Farkas [10], Stranski and Kaischew [11], Becker and Döring [12], Volmer [13], Frenkel [14], Fisher and Turnbull [15], and others. Thermodynamic aspects of CNT are described by the fundamental approach to the description of heterogeneous systems developed by J. W. Gibbs [16,17] originally in application to condensation and boiling. Clusters of the newly evolving phase are assumed to be of spherical shape and surface contributions to the thermodynamic quantities are described by the surface tension. The theoretical foundation of the possibility to treat critical crystal nucleation (critical crystals may have, in general, quite different shapes and the relations describing the surface contributions to the thermodynamic potentials are, in general, much more complex) in terms of such simplified model is described in detail in [18,19]. This model we will use in the present study.

Utilizing CNT, the expression for the steady-state nucleation rate, J(T,p), is commonly written in the form [4]

(1) $$J\left(T,p\right)=J_{0}\exp\left(-\frac{W_{c}}{k_{B}T}\right)\;,\;J_{0}=c\sqrt{\frac{\sigma }{k_{B}T}}\left(\frac{D}{d_{0}}\right)≅c\frac{\sqrt{\sigma k_{B}T}}{\eta d_{0}^{2}}\;,$$

(2) $$W_{c}=\frac{16\pi }{3}\frac{\sigma^{3}}{{\left(\Delta g\right)}^{2}}\;,\;R_{c}=\frac{2\sigma }{\Delta g}\;.$$

Here, $W_{c}$ is the work of critical cluster formation, $k_{B}$ is the Boltzmann constant, and *T* is the absolute temperature. Assuming a spherical shape (and choosing, as it is commonly done in applications to nucleation, the surface of tension as the dividing surface [16,17]), the surface area of the critical cluster is given by $A_{c}=4\pi R_{c}^{2}$, where $R_{c}$ is the critical cluster radius and σ is the surface tension both taken for this particular dividing surface.

The basic concepts of Gibbs’ thermodynamic theory of heterogeneous systems, used in derivation of Equation (2), are also employed in the generalization of Gibbs’ classical treatment performed by us [20,21,22,23]. Being aware of possible different opinions concerning the correct form of Equation (2) (denoted commonly as “Renninger–Wilemski problem” [24]), we would like to underline that Equation (2) hold—as far as the surface of tension is chosen as the dividing surface as we do—both in cases when the surface tension is taken as a constant involving the capillarity approximation and similarly also when a curvature dependence of the surface tension is accounted for [16,17,25,26,27,28,29]. The critical cluster size is determined in such approach by the ratio of surface tension, σ, and the thermodynamic driving force of crystallization, Δg, the change of the bulk contributions to the Gibbs’ free energy per unit volume of the crystal phase both for one-component and multi-component systems [4,30].

In applications of CNT to crystal nucleation, the thermodynamic driving force of crystallization is specified in accordance with Gibbs’ theory of surface phenomena. It has the same value for both nucleation and growth processes, i.e., the possibility that critical clusters have properties different from the properties of the evolving crystalline phase is, as a rule, not accounted for. Corrections in the theoretical description of crystal nucleation rates are introduced in CNT into the description exclusively via curvature corrections to the surface tension [18,19]. In contrast, employing the generalized Gibbs approach [4,21,22,23], the bulk properties of the critical clusters are also shown to deviate, as a rule, significantly from the properties of the newly evolving macroscopic phases and can be appropriately accounted for.

While the exponential term in Equation (1) is determined by thermodynamic aspects of nucleation, the pre-exponential factor, $J_{0}$, in this relation is governed by the kinetic mechanisms underlying the aggregation processes. The expressions given in Equation (1) have been advanced originally for the case of homogeneous crystallization of one-component liquids. These liquids are characterized by a particle density, $c=1/d_{0}^{3}$, where $d_{0}$ is a measure of the size of the ambient phase particles, and *D* is their diffusion coefficient in the liquid. Since the relevant for nucleation and growth diffusion coefficients are frequently not known, they are replaced in applications of CNT to crystallization by the Newtonian viscosity, η. This replacement is performed in terms of the Stokes–Einstein–Eyring relation, $D≅k_{B}T/\eta d_{0}$. Although formulated originally in application to one-component systems, Equation (1) can be employed also in more general and more realistic cases, as discussed in detail in [4,31,32]. In particular, for multi-component systems nucleation may be limited not kinetically but by diffusion processes of the different components or by some intermediate form of aggregation kinetics (see also [33]). Consequently, in such cases the parameter $d_{0}$ may become dependent on the size of the critical clusters.

For one-component systems we can perform the substitution $\Delta g=\Delta \mu /v_{\alpha }$, where Δμ is the difference between the chemical potential per particle in the liquid and the crystal phases and $v_{\alpha }≅d_{0}^{3}$ is the volume per particle in the crystal phase. Utilizing these notations, we obtain approximately

(3) $$\frac{dW_{c}}{d(\Delta \mu )}=-n_{c}\;.$$

Here, $n_{c}$ is the number of particles in the critical cluster. The assumptions employed in the derivation of Equation (3) will be specified below. One of them consists in the application of the capillarity approximation, i.e., the surface tension is assumed to be equal to the respective value for an equilibrium coexistence of liquid and crystal at planar interfaces.

Relations of the form of Equation (3) are widely discussed in the literature [3,4,16,21,34,35,36,37,38,39,40,41,42,43,44,45,46,47,48,49,50,51], starting immediately with the work by Gibbs. They are denoted, following Kashchiev [36], as the first nucleation theorem. The interest in such relations is caused by the fact that they allow one to derive conclusions on the properties of critical clusters in nucleation based on measurements of the nucleation rate. Indeed, in line with Equation (3), variations of the nucleation rate are intrinsically connected with changes of the work of critical cluster formation. Consequently, from measurements of the nucleation rate one can make conclusions on the change of the work of critical cluster formation caused by variations of the external control parameters and, utilizing relations of the form of Equation (3), on the properties of critical clusters. Of course, in such procedures the variations of the kinetic coefficients like diffusion coefficient or viscosity have to also be appropriately accounted for (see, e.g., [3,46]). Most studies of applications of the nucleation theorem are devoted to condensation. Here we concentrate our attention on crystal nucleation utilizing appropriate approximations valid for this particular case of phase formation (for more general approaches and the discussion of different forms of the nucleation theorem, see, e.g., [40,42,43,44]).

It was stressed by Kashchiev and Oxtoby [36,40] that relations of the form of Equation (3) may hold independently on the method of how surface correction terms are introduced into the expression for the change of the Gibbs free energy. However, once such surface corrections terms may be of significance, then their proper specification is of huge importance for a correct application of the nucleation theorem to the determination of the properties of critical clusters (see, e.g., [42,43]). As noted in another context by Albert Einstein in conversation with Werner Heisenberg: *“Only the theory decides what one can observe”* [52]. In our case, this statement refers to the question how one can determine the properties of critical clusters from the derivatives of the work of critical cluster formation by the thermodynamic driving force or other appropriate control parameters specifying theoretically correctly both the bulk and, in particular, the surface contributions to critical cluster formation.

In line with the above given considerations, in the present paper, we derive several versions of Equation (3) utilizing different approaches in the specification of both the bulk and surface contributions to the work of critical cluster formation. We start the analysis first taking as granted the assumptions commonly employed in CNT (Section 2.1) assuming, in particular, that the expressions for the thermodynamic driving force employed are widely correct. We perform here the analysis immediately for the general case of crystal nucleation in multi-component systems going beyond the case of one-component systems as studied in [36] and extending the results obtained by us earlier in [42]. Once the expressions for the thermodynamic driving force are considered to be widely correct, the only method which can be used in CNT in order to reconcile experimental data with theoretical predictions consists in the introduction of a size dependence of the surface tension [16,21,53,54,55,56] of the critical clusters (or, equivalently, a dependence of the surface tension on pressure and temperature determining the degree of metastability of the melt, respectively, the thermodynamic driving force). Based on the Stefan–Skapski–Turnbull rule, in recent papers [18,19,30,57,58] expressions for the dependence of the surface tension on both external control parameters have been derived. These relations are utilized in the present analysis to arrive at expressions for the derivatives of the work of critical cluster formation with respect to the thermodynamic driving force. In experiments, the thermodynamic driving force can be varied by changes of composition, pressure, and/or temperature. By this reason, we will formulate dependencies of the form of Equation (3) also for these particular cases (Section 2.2). Our approach is compared with the method employed by Kashchiev [36] in order to arrive at expressions of the form of Equation (3). It is shown that, provided the underlying assumptions hold, the approach developed by Kashchiev is valid also for multi-component systems (Section 2.3).

In a second step of the present analysis, an extension of Equation (3) is derived for the case that the work of critical cluster formation is described in terms of the generalized Gibbs approach not utilizing a variety of assumptions inherent in CNT and, in particular, accounting appropriately for the dependence of the bulk properties of critical crystallites on the degree of deviation from thermodynamic equilibrium. As will be shown, also for such a general situation, a formulation of the nucleation theorem in application to crystal nucleation can be given (Section 3) which is widely similar to the result obtained by us in the present paper in terms of CNT. A summary of the results and their brief discussion completes the paper (Section 4).

## 2. Nucleation Theorem: Analysis in Terms of Classical Nucleation Theory

### 2.1. Basic Equations and Results

In CNT, the thermodynamic driving force of crystallization, Δg, is expressed commonly in a first approximation via the difference of the bulk contributions to the Gibbs free energy between liquid and crystal per unit volume of the crystal phase [3,4,19,30] both taken at the pressure, *p*, and temperature, *T*, of the liquid phase, i.e., $\Delta g≅\Delta g_{CNT}\left(T,p\right)$ with

(4) $$\Delta g_{CNT}\left(T,p\right)=\sum_{i=1}^{k}\rho_{i\alpha }\left(\mu_{i\beta }\left(T,p,\{x_{i\beta }\}\right)-\mu_{i\alpha }\left(T,p,\{x_{i\alpha }\}\right)\right)\;.$$

Here, $\mu_{i\alpha }$ and $\mu_{i\beta }$ are the chemical potentials per particle of the different components in the crystal (specified by the subscript α) and the liquid (specified by the subscript β) phases, $x_{i\alpha /\beta }$ and $\rho_{i\alpha /\beta }$ are the molar fractions and particle densities of the different components in the respective phases. In the derivation of this relation it is assumed that the composition of the critical crystallites is widely identical to the composition of the newly evolving macroscopic phases [4,30]. Consequently, the thermodynamic driving force is essentially a function of pressure and temperature.

Instead of Δg, frequently the chemical potential difference, Δμ, is also utilized for the description of the thermodynamic driving force being for multi-component systems of the form [4,30]

(5) $$\Delta \mu \left(T,p\right)=\sum_{i=1}^{k}x_{i\alpha }\left(\mu_{i\beta }\left(T,p,\{x_{i\beta }\}\right)-\mu_{i\alpha }\left(T,p,\{x_{i\alpha }\}\right)\right)\;,$$

(6) $$\Delta g_{CNT}\left(T,p\right)=\frac{1}{v_{\alpha }}\Delta \mu \left(T,p\right)\;,\;x_{i\alpha }=\frac{n_{i\alpha }}{n}\;,\;v_{\alpha }=\frac{V_{\alpha }}{n}\;.$$

Here, *n* is the total number of particles and $n_{i\alpha }$ are the numbers of particles of the *i*-th component, $x_{i\alpha }$ are its molar fractions and $v_{\alpha }$ the (average) volume per particle all of them referring to the crystal phase, $\left\{x_{i\beta }\right\}$ is the set of independent molar fractions of the different components in the liquid phase. Employing these notations, Equation (2) can be reformulated as

(7) $$W_{c}=\frac{16\pi }{3}\frac{\sigma^{3}v_{\alpha }^{2}}{{\left(\Delta \mu \right)}^{2}}\;,\;R_{c}=\frac{2\sigma v_{\alpha }}{\Delta \mu }\;,$$

and supplemented by a relation for the number of particles, $n_{c}$, in the critical cluster

(8) $$n_{c}=\frac{32\pi }{3}{\left(\frac{\sigma }{\Delta \mu }\right)}^{3}v_{\alpha }^{2}\;.$$

By taking the derivative of $W_{c}$ with respect to Δμ, we obtain then

(9) $$\frac{dW_{c}}{d(\Delta \mu )}=-n_{c}\left(1-\frac{3}{2}\frac{d\ln\sigma }{d\ln(\Delta \mu )}\right)\;.$$

Utilizing the capillarity approximation (assuming the surface tension to be equal to the respective value for a planar equilibrium coexistence of liquid and crystal phases at a planar interface, i.e., that it does not change in response on variations of the degree of deviation from equilibrium, Δμ), Equation (9) is reduced to the particular form given by Equation (3) but here referring to nucleation in multi-component systems.

### 2.2. Some Alternative Forms of the Nucleation Theorem

Equation (9) connects variations of the work of critical cluster formation in dependence on the thermodynamic driving force of the phase transformation, expressed here via Δμ(T,p), with changes of the surface tension caused by such variations of the thermodynamic driving force. In order to derive particular expressions for the application of this relation to experiment, both the expressions for the thermodynamic driving force and the surface tension have to be specified expressing them via those control parameters, which can be varied in experiment. Such specification will be performed here again for the general case of crystal nucleation in multi-component systems.

Equation (4) is the most general expression for the description of the thermodynamic driving force of crystallization in CNT. However, detailed knowledge of the chemical potentials of the different components in both phases is commonly not available. By this reasoning, this general relation is transformed into expressions allowing one to compute Δg(T,p) directly by knowing the parameters ($T_{m},p_{m}$) of some particular macroscopic equilibrium state liquid-crystal and the respective deviations of temperature, $\left(T-T_{m}\right)$, and pressure, $\left(p-p_{m}\right)$, from it. Following such ideas, the dependence of thermodynamic driving force of crystallization on temperature and pressure can be described in a good approximation in the form (for details see [4,19,30,58])

(10) $$\Delta g_{CNT}\left(T,p\right)≅\Delta h_{m}\left(1-\frac{T}{T_{m}}\right)\left[1-\frac{\Delta c_{p}\left(T_{m},p_{m}\right)}{2\Delta s_{m}}\left(1-\frac{T}{T_{m}}\right)\right]$$

$$+p_{m}\Delta v_{m}\left(\frac{p}{p_{m}}-1\right)\left[1-\frac{p_{m}\Delta \kappa_{T}\left(T_{m},p_{m}\right)}{2\Delta v_{m}}\left(\frac{p}{p_{m}}-1\right)\right]\;.$$

Here, $\Delta h_{m}$ and $\Delta s_{m}$ are the melting enthalpy and melting entropy per unit volume of the crystal at the temperature, $T_{m}$, and the pressure, $p_{m}$, $\Delta v_{m}=\left(V_{liquid}-V_{crystal}\right)/V_{crystal}$, $\Delta c_{p}$ and $\Delta \kappa_{T}$ are the differences of specific volume, specific heat, and isothermal compressibility also taken at $\left(T_{m},p_{m}\right)$. These relations can be employed for both one- and multi-component (considered here) systems and both stoichiometric and non-stoichiometric crystallization. As a rule, the inequalities $\Delta h_{m}>0$ and $\Delta v_{m}>0$ hold. However, for some substances like water, crystallization may be accompanied by an increase of the volume resulting in $\Delta v_{m}<0$. In such cases, a decrease of pressure is required in order to initiate crystal nucleation. These phenomena are discussed in detail in an accompanying paper [59] (see also [60]).

Similarly to the thermodynamic driving force, the dependence of the surface tension on temperature and pressure has to be known for the determination of the work of critical cluster formation, Equation (2). This dependence can be written as [19,57,58]

(11) $$\frac{\sigma (T,p)}{\sigma (T_{m},p_{m})}≅\left(\frac{T}{T_{m}}\right)\left[1-\frac{\Delta c_{p}}{\Delta s_{m}}\left(1-\frac{T}{T_{m}}\right)-\frac{p_{m}\Delta \alpha_{T}}{\Delta s_{m}}\left(\frac{p}{p_{m}}-1\right)\right]\;.$$

Here, $\Delta \alpha_{T}$ is the difference in the thermal expansion coefficients between liquid and crystal. A combination of Equations (9)–(11) allows us to determine the change of the work of critical cluster formation in dependence on either pressure or temperature. The respective computations are straightforward and by this reason not given here in the general form. Anyway, to have some impression below two special cases are considered employing truncated expressions for the dependencies of the thermodynamic driving force and the surface tension.

In case the chemical potential difference and surface tension is caused by temperature variations described in such first-order approximation as

(12) $$\Delta \mu \left(T,p_{m}\right)≅v_{\alpha }\Delta h_{m}\left(1-\frac{T}{T_{m}}\right)\;,\;\sigma \left(T,p_{m}\right)≅\sigma \left(T_{m},p_{m}\right)\left(\frac{T}{T_{m}}\right)\;,$$

Equation (9) yields

(13) $$\frac{dW_{c}}{dT}≅n_{c}\left(\frac{3v_{\alpha }\Delta h_{m}}{2T}\right)\left(1-\frac{T}{3T_{m}}\right)\;.$$

For pressure-induced nucleation, using the approximations,

(14) $$\Delta \mu \left(T_{m},p\right)≅v_{\alpha }p_{m}\Delta v_{m}\left(\frac{p}{p_{m}}-1\right)\;,$$

$$\sigma \left(T_{m},p\right)≅\sigma \left(T_{m},p_{m}\right)\left[1-\left(\frac{p_{m}\Delta \alpha_{T}}{\Delta s_{m}}\right)\left(\frac{p}{p_{m}}-1\right)\right]\;,$$

we obtain instead

(15) $$\frac{dW_{c}}{dp}≅-n_{c}\frac{v_{\alpha }\Delta v_{m}}{2}\left\{\frac{2+\left(\frac{p_{m}\Delta \alpha_{T}}{\Delta s_{m}}\right)\left(\frac{p}{p_{m}}-1\right)}{1-\left(\frac{p_{m}\Delta \alpha_{T}}{\Delta s_{m}}\right)\left(\frac{p}{p_{m}}-1\right)}\right\}$$

both as zeroth-order estimates. As evident, in both cases the partial derivatives of the work of critical cluster formation taken with respect to the external control parameters are proportional to the number of particles in the critical clusters. However, in these cases the coefficient of proportionality is generally quite different from one.

As noted above, in CNT the bulk state parameters of the critical crystallites are assumed to be widely identical to the properties of the newly evolving macroscopic phases. Consequently, in line with Equation (5), the thermodynamic driving force can be considered as a function of the set of variations of the chemical potential differences, $\left\{\Delta \mu_{i}\right\}$, of the different components. Provided it would be possible to vary only one of the chemical potential differences, say $\Delta \mu_{j}$, then (see, e.g., also [50,51])

(16) $$\frac{\partial W_{c}}{\partial (\Delta \mu_{j})}=\frac{dW_{c}}{d(\Delta \mu )}\frac{\partial \Delta \mu }{\partial (\Delta \mu_{j})}=-n_{jc}\left(1-\frac{3}{2}\frac{d\ln\sigma }{d\ln(\Delta \mu )}\right)\;,\;n_{jc}=n_{c}x_{j\alpha }\;.$$

Here, $n_{jc}$ is the number of particles of the *j*-th component in the critical cluster. However, in any attempts to realize such variations in experiment, one has to take care about possible limitations of the applicability of these equations caused by necessity to fulfil the Gibbs–Duhem relations [42,43,61].

In contrast to $\Delta \mu_{j}$, the independent molar fractions, $x_{j\beta }$, j=1,2,…,(k−1), of the liquid phase can be varied separately. Assuming that such process of variation of only one independent molar fraction is performed at constant pressure and temperature, we obtain

(17) $$\frac{\partial W_{c}}{\partial x_{j\beta }}=\frac{dW_{c}}{d(\Delta \mu )}\frac{\partial \Delta \mu }{\partial x_{j\beta }}$$

or

(18) $$\frac{\partial W_{c}}{\partial x_{j\beta }}=-n_{c}\left(1-\frac{3}{2}\frac{d\ln\sigma }{d\ln(\Delta \mu )}\right)\frac{\partial }{\partial x_{j\beta }}\left\{\sum_{i=1}^{k}x_{i\alpha }\left(\mu_{i\beta }\left(T,p,\{x_{i\beta }\}\right)-\mu_{i\alpha }\left(T,p,\{x_{i\alpha }\}\right)\right)\right\}\;.$$

A change of the composition of the liquid may lead to variations of the composition of the crystal phase coexisting in equilibrium with the liquid. If such variations of the composition of the crystalline phase can be neglected, we obtain as a special case

(19) $$\frac{\partial W_{c}}{\partial x_{j\beta }}=-n_{c}\left(1-\frac{3}{2}\frac{d\ln\sigma }{d\ln(\Delta \mu )}\right)\sum_{i=1}^{k}x_{i\alpha }\left(\frac{\partial \mu_{i\beta }\left(T,p,\{x_{i\beta }\}\right)}{\partial x_{j\beta }}\right)\;.$$

Again, the partial derivative $\left(\partial W_{c}/\partial x_{j\beta }\right)$ is proportional to the critical cluster size, $n_{c}$, but the evaluation of the coefficient of proportionality requires a detailed knowledge both on the bulk properties of the coexisting phases and the change of the surface tension with respect to variations of the thermodynamic driving force for this particular method of variation of the state of the liquid.

### 2.3. Comparison with the Approach Employed by Kashchiev

In his first paper ([36], see also [3,40]), Kashchiev considered exclusively one-component systems. He formulated an equation for the work of critical cluster formation of the form of Equation (7) for this particular case. He assumed then validity of the capillarity approximation and arrived at Equation (3). As shown above, this method is similarly applicable to the description of crystal nucleation for the general case of multi-component systems and accounting for a dependence of the surface tension of critical crystal nuclei immediately yields the generalization given by Equation (9).

The account of surface free energy contributions was performed by Kashchiev in a different way as compared to the standard one used here. In line with Gibbs’ thermodynamic approach and the approximations usually employed in the description of crystal nucleation, the bulk contribution to the change of the Gibbs’ free energy was written as −nΔμ supplemented but surface energy terms given by F(n,Δμ). Accounting for both bulk and surface contributions, the work of formation of a cluster containing *n* particles is expressed then as [36]

(20) W(n,Δμ)=−nΔμ+F(n,Δμ).

It is stated that the derivative of the work of critical cluster formation with respect to Δμ reads

(21) $$\frac{dW_{c}}{d(\Delta \mu )}=-n_{c}+\frac{\partial F_{c}}{\partial (\Delta \mu )}\;,$$

denoting this expression as nucleation theorem. In the second term on the right hand side of Equation (21), the partial derivative with respect to Δμ implies that the derivative has to be taken at constant values of the cluster size.

Utilizing Gibbs’ method and certain approximations, one obtains as a special case F(n,Δμ)≅σA then. Here, $A=4\pi R^{2}$ is the surface area of the crystallite. A substitution of this relation into Equation (21) leads immediately to Equations (7) and (9), again, when Δμ is expressed via Equation (5). Consequently, in contrast to the respective statements in [36,40], Equations (20) and (21) hold not only for one- but also for multi-component systems as far as the assumptions commonly utilized in CNT are fulfilled. Such extension of the approach formulated by Kashchiev [36] to multi-component systems was also performed already by Yang Gao et al. in [50,51].

Although both methods lead for this particular case to the same results, we consider our approach to be preferable. It involves exclusively the analysis of parameters of critical clusters and does not require the specification of the bulk and surface contributions to cluster formation for arbitrary cluster sizes as it has to be done in the formulation of Equation (20). If this procedure is realized in a general form, very detailed considerations have to be performed, as discussed in detail in [20,21,22,62,63]. Moreover, it is supposed in the formulation of Equation (20) that the surface contributions are a function of the number of particles in the cluster, *n*, and of the driving force, Δμ. In general, this is as a rule not the case as evident from Gibbs’ theory of heterogeneous systems [16,17]. According to Gibbs’ adsorption equation, the surface tension of critical clusters is a function of either the set of state parameters of the newly evolving or of the ambient phase. For example, as evident from Equation (11), the surface tension of critical clusters can be expressed as a function of pressure and temperature. In line with Equation (10), one and the same values of Δμ may yield, in principle, different values of the surface tension. Moreover, utilizing exclusively Gibbs’ theory (referring to thermodynamic equilibrium states) nothing can be said concerning the value of the surface tension for clusters not being in equilibrium with the ambient liquid.

In our approach, it is merely required that infinitesimal variations of the state parameters of the ambient phase like $\left(T,p,\left\{x_{i\beta }\right\}\right)$ lead to infinitesimal variations of $W_{c}$, Δμ, and σ (referred also exclusively to the critical cluster) and that the differential quotient, (dσ/d(Δμ)), has well-defined values. This difference in the interpretation is even more important if the expressions for the thermodynamic driving force and the surface tension are more complex as compared with the relations formulated based on the assumptions commonly employed in CNT and underlying also Equation (20). The respective generalizations and their consequences will be analyzed in the next section.

## 3. Thermodynamics of Cluster Formation: Beyond the Classical Gibbs’ Approach

### 3.1. One Main Deficiency of Classical Nucleation Theory

As noted, for example, by John von Neumann [64], a theory has to fulfil two demands, it has *“correctly to describe phenomena from a reasonably wide area. Furthermore, it must satisfy certain esthetic criteria—that is, in relation to how much it describes, it must be rather simple”*. In CNT, the possibility to describe in a relatively simple way experimental data on crystal nucleation is related to the assumption that the bulk properties of the critical clusters are widely identical to the properties of the newly evolving macroscopic phases. Once the critical clusters have such properties, also sub- and super-critical clusters can be considered to have the same properties.

However, already in the course of developing the basic concepts of CNT it has been understood clearly that the bulk properties of the critical (and, consequently, of sub- and super-critical) crystallites may also be quite different as compared to the properties of the newly evolving macroscopic phases (see, e.g., [4,7,65,66,67,68]). As a consequence, alternative continuums approaches to the description of heterogeneous systems have been developed and applied to the description of phase formation processes [69,70,71], repeating and advancing the work performed much earlier by J. D. van der Waals [72,73]. These theoretical methods and their generalization, as well as computer simulations, confirm in line with an increasing number of experimental studies the point of view that the parameters of the critical cluster may differ significantly from the properties of the evolving macroscopic phases (e.g., [74,75,76,77,78,79,80,81,82]). Consequently, the computation of the thermodynamic driving force performed under the assumption of nearly constant macroscopic values of the bulk state parameters of the crystal clusters can be only an approximation. In some cases, this assumption may appropriately reflect the kinetics of crystallization for some selected systems. As a rule, however, it may lead to severe inconsistencies in the description of the experimental data.

The mentioned basic assumption of CNT is supported by Gibbs’ classical method of description of heterogeneous systems. Consequently, the question arises whether one has to abandon this approach at all. However, as shown by us these problems can be resolved by developing a generalization of the classical Gibbs approach [4,21,22,23,83,84,85,86]. As a first step in such a generalization, we developed a theory of heterogeneous systems for non-equilibrium states. This is a straightforward generalization of Gibbs’ classical approach, who restricted his considerations exclusively to equilibrium states. It accounts, in particular, appropriately for the fact that in non-equilibrium states the surface tension has to depend, in general, on the state parameters of both the ambient and newly evolving phases.

In order to determine the thermodynamic potentials for non-equilibrium states, one of the problems one has to solve consists in the determination of the values of the chemical potentials, $\mu_{j\sigma }$, and the temperature, $T_{\sigma }$, referring to the surface phase. In accordance with an intensive analysis of such problems by Defay et al. [87], Prigogine and Bellemans [88] (*“a surface phase has no real autonomy, in general”*), and Rowlinson and Widom [89] (*“we cannot measure or define unambiguously the thermodynamic properties of the surface phase”*) we postulated [20,21,22] that the superficial surface parameters, $T_{\sigma }$ and $\mu_{j\sigma }$, have to be set equal to the respective quantities of the ambient phase

(22) $$T_{\sigma }=T_{\beta }\;,\;\mu_{j\sigma }=\mu_{j\beta }\;.$$

As one consequence, in above equations, the superficial particle numbers, $n_{i\sigma }$, and the superficial entropy, $S_{\sigma }$, which enter the description in terms of Gibbs theory as correction terms describing effects of the interface, are included into the number of particles of the newly evolving phase, $n_{i\alpha }=\rho_{i\alpha }V_{\alpha }$, and the volume density of the entropy, $s_{\alpha }$, of this phase, correspondingly.

Based on such generalization, more complex equations for the description of the properties of the critical clusters governing nucleation are obtained as compared to Gibbs’ classical treatment. Accounting for such effects and the described method of their description, we will now advance a reformulation of the nucleation theorem.

### 3.2. Basic Equations and Results

Both in the classical and generalized Gibbs’ approaches [16,20,21,22,23,23], the work of critical cluster formation, $W_{c}$, and the critical cluster radius, $R_{c}$ (referred to the surface of tension), can be expressed as

(23) $$W_{c}=\frac{16\pi }{3}\frac{\sigma^{3}\left(p_{\alpha },T_{\alpha },\left\{x_{i\alpha }\right\};p_{\beta },T_{\beta },\left\{x_{i\beta }\right\}\right)}{{\left(\Delta g(p_{\alpha },T_{\alpha },\left\{x_{i\alpha }\right\};p_{\beta },T_{\beta },\left\{x_{i\beta }\right\})\right)}^{2}}\;,$$

(24) $$R_{c}=\frac{2\sigma (p_{\alpha },T_{\alpha },\left\{x_{i\alpha }\right\};p_{\beta },T_{\beta },\left\{x_{i\beta }\right\})}{\Delta g(p_{\alpha },T_{\alpha },\left\{x_{i\alpha }\right\};p_{\beta },T_{\beta },\left\{x_{i\beta }\right\})}\;.$$

The thermodynamic driving force of cluster formation is given generally by [29,30]

(25) $$\Delta g\left(p_{\alpha },T_{\alpha },\left\{x_{i\alpha }\right\};p_{\beta },T_{\beta },\left\{x_{i\beta }\right\}\right)=s_{\alpha }\left(T_{\beta }-T_{\alpha }\right)+\left(p_{\alpha }-p_{\beta }\right)$$

$$+\sum_{i=1}^{k}\rho_{i\alpha }\left(\mu_{i\beta }\left(p_{\beta },T_{\beta },\left\{x_{i\beta }\right\}\right)-\mu_{i\alpha }\left(p_{\alpha },T_{\alpha },\left\{x_{i\alpha }\right\}\right)\right)\;.$$

In the derivation of these relations, the fundamental equation of Gibbs’ approach was generalized to allow the surface tension to be a function of the state parameters of both ambient and newly evolving phases.

Equation (4), employed commonly in CNT, is obtained as a limiting case from Equation (25) provided that temperature and pressure of the critical cluster and the ambient liquid coincide. The first of these assumptions ($T_{\alpha }\to T_{\beta }$) is in agreement with the equilibrium conditions

(26) $$\mu_{i\beta }\left(p_{\beta },T_{\beta },\left\{x_{j\beta }\right\}\right)-\mu_{i\alpha }\left(p_{\alpha },T_{\alpha },\left\{x_{j\alpha }\right\}\right)=0\;\mathrm{for}\;i=1,2,\ldots ,k\;,$$

(27) $$T_{\beta }-T_{\alpha }=0\;,$$

(28) $$p_{\beta }-p_{\alpha }+\frac{2\sigma }{R_{c}}=0\;,$$

as obtained in the classical Gibbs approach [16,17], while the second ($p_{\alpha }\to p_{\beta }$), in general, is not. Approximately, Equation (4) can be obtained from Equation (25) by a truncated Taylor expansion [4,30]. In this procedure it is assumed that the bulk state parameters of the critical clusters depend neither on pressure nor on temperature. The conditions $T_{\alpha }\to T_{\beta }$ and $p_{\alpha }\to p_{\beta }$ are also employed in the derivation of Equations (8) and (11) for the dependence of the specific surface energy on temperature and pressure.

In the framework of the generalized Gibbs approach, the critical cluster size is determined by Equation (24) in combination with Equation (25), again. Moreover, instead of Equations (26) and (27) we get the additional equilibrium conditions now in the form [4,21,22,23,29],

(29) $$\mu_{i\beta }-\mu_{i\alpha }=\frac{3}{R_{c}}{\left(\frac{\partial \sigma }{\partial \rho_{i\alpha }}\right)}_{\left\{\rho_{i\beta }\right\},s_{\beta }}\;\mathrm{for}\;i=1,2,\ldots ,k\;,$$

(30) $$T_{\beta }-T_{\alpha }=\frac{3}{R_{c}}{\left(\frac{\partial \sigma }{\partial s_{\alpha }}\right)}_{\left\{\rho_{i\beta }\right\},s_{\beta }}\;.$$

Both the equilibrium conditions for pressure and also for thermal and diffusion equilibrium are modified. In particular, we conclude that the first of the assumptions employed in CNT ($T_{\alpha }=T_{\beta }$) for the determination of the thermodynamic driving force also also lacks any theoretical foundation (see also [90,91]). Note further that in this more general situation (described by Equations (29) and (30)), Equation (24) is not reduced to Equation (28) as this is the case in Gibbs’ classical approach.

It follows as an additional consequence that the bulk parameters of the critical clusters may vary considerably in dependence on the degree of metastability. This effect is determined by the derivatives of the surface tension with respect to the state parameters of the cluster phase. In the generalized Gibbs’ approach, the surface tension is a function of the state parameters of both ambient and newly evolving phases. By this reason, here derivatives of the surface tension occur in the equilibrium conditions. Indeed, utilizing Equations (29) and (30), the correct expression, Equation (25), for the thermodynamic driving force of nucleation can be expressed as

(31) $$\Delta g=\left(p_{\alpha }-p_{\beta }\right)+\frac{3}{R_{c}}\left[s_{\alpha }{\left(\frac{\partial \sigma }{\partial s_{\alpha }}\right)}_{\left\{\rho_{j\beta }\right\},s_{\beta }}+\sum_{i=1}^{k}\rho_{i\alpha }{\left(\frac{\partial \sigma }{\partial \rho_{i\alpha }}\right)}_{\left\{\rho_{j\beta }\right\},s_{\beta }}\right]\;.$$

Variations of the bulk state parameters of the cluster phase in response to variations of the degree of metastability may be, consequently, of considerable effect concerning the value of the thermodynamic driving force.

In order to employ above derived equations, we have to know the surface tension (and here in an even more complex form as compared to the classical approach) as a function of the state parameters of both coexisting phases. An expression for the surface tension can be obtained, again, via the Stefan–Skapski–Turnbull relation. We may write [57,58]

(32) $$\frac{\sigma }{\sigma (T_{m},p_{m})}=\frac{1}{T_{m}\Delta s_{m}}\left\{T_{\beta }s_{\beta }-T_{\alpha }s_{\alpha }+\sum_{i=1}^{k}\rho_{i\alpha }\left(\mu_{i\beta }-\mu_{i\alpha }\right)\right\}\;.$$

This expression allows us to determine the dependence of the surface tension on pressure and temperature, again. However, in contrast to Equation (11), here the dependence of the bulk state parameters of the critical clusters on pressure and temperature has to also be accounted for. These state parameters have to be determined by the equilibrium conditions Equations (29) and (30), again. Substituting the equilibrium conditions into Equation (32), we get instead of Equation (8)

(33) $$\frac{\sigma }{\sigma (T_{m},p_{m})}=\frac{1}{T_{m}\Delta s_{m}}\left\{T_{\beta }\left(s_{\beta }-s_{\alpha }\right)+\frac{3}{R_{c}}\left[s_{\alpha }{\left(\frac{\partial \sigma }{\partial s_{\alpha }}\right)}_{\left\{\rho_{j\beta }\right\},s_{\beta }}+\sum_{i=1}^{k}\rho_{i\alpha }{\left(\frac{\partial \sigma }{\partial \rho_{i\alpha }}\right)}_{\left\{\rho_{j\beta }\right\},s_{\beta }}\right]\right\}$$

(for details see [57]).

The specification of the dependence of the thermodynamic driving force and the surface tension, accounting for possible variations of both bulk and surface state parameters of the critical clusters, is, consequently, a highly complex task (see also [23]). For any set of state parameters of the ambient liquid phase $\left(p_{\beta },T_{\beta },\left\{x_{i\beta }\right\}\right)$, first the state parameters of the critical clusters have to be determined. This procedure can be performed via the equilibrium conditions, Equations (24), (29) and (30), supplying us with (k+2) relations for the determination of the (k+1) bulk state parameters and the size of the critical cluster. Having at one’s disposal these parameters, both the surface tension and the thermodynamic driving force can be computed. Infinitesimal variations of the state parameters of the ambient liquid phase will result, consequently, in similar variations of both the thermodynamic driving force, dΔg, and the surface tension, dσ. For the formulation of the general form of the nucleation theorem employing the generalized Gibbs’ approach only latter mentioned conclusion is of importance, i.e., that the ratio (dσ/d(Δg)) is well-defined.

Indeed, as noted, the work of critical cluster formation is given also in the generalized Gibbs’ approach by the simple relation, Equation (23). Taking the derivative with respect to Δg, we obtain similarly to Equations (8) and (9)

(34) $$V_{c}=\frac{32\pi }{3}{\left(\frac{\sigma }{\Delta g}\right)}^{3}\;,$$

(35) $$\frac{dW_{c}}{d(\Delta g)}=-V_{c}\left(1-\frac{3}{2}\frac{d\ln\sigma }{d\ln(\Delta g)}\right)\;.$$

Here, $V_{c}$ is the volume of the critical cluster. The general form of the nucleation theorem remains the same as derived in employing basic assumptions of CNT. Of course, introducing the assumptions of CNT at this stage, Equation (9) is reproduced as a special limiting case, again.

Note, however, that also another expression derived in terms of Gibbs classical approach can be obtained based on Equation (35) (but not via Equation (9) involving additional approximations sketched here earlier) as a special case. Utilizing Gibbs’ equilibrium conditions, Equations (26) and (27), Δg can be replaced by $\left(p_{\alpha }-p_{\beta }\right)$. In such a limiting case, we obtain

(36) $$\frac{dW_{c}}{d(p_{\alpha }-p_{\beta })}=-V_{c}\left(1-\frac{3}{R_{c}}\frac{d\sigma }{d(p_{\alpha }-p_{\beta })}\right)$$

derived by us earlier in [42] utilizing Gibbs’ classical approach [16].

Although the nucleation theorem is of very similar form to the one derived in terms of CNT, the specification of the conclusions of what its consequences are when, e.g., either composition, pressure, or temperature are varied becomes a much more complex task. Consequently, the predictions concerning the parameters of the critical clusters as obtained via the generalized Gibbs approach may be quite different from the conclusions obtained in terms of CNT (see also [42]).

## 4. Results and Discussion

In the first part of the present analysis of the nucleation theorem, the main ideas of CNT are utilized. The results can be summarized as follows: (i) By employing the classical Gibbs’ approach in the thermodynamic description of heterogeneous systems and the basic assumptions of classical nucleation theory, a general form of the nucleation theorem, Equation (9), can be formulated valid not only for one-component but generally for multi-component systems. (ii) This result is taken then as the starting point for the derivation of particular forms, Equations (13), (15), (16) and (18) of this theorem for the cases that the degree of deviation from equilibrium is caused by variations of either composition of the liquid phase, temperature, and/or pressure. In this procedure, the expressions mentioned above for the curvature dependence of the surface tension, respectively, on pressure and/or temperature are utilized. (iii) It is shown that the nucleation theorem in the form of Equation (21) as expressed by Kashchiev [36] can be extended in application to crystallization to multi-component systems, provided the basic assumptions commonly employed in classical nucleation theory are fulfilled.

In the second part of the analysis, effects caused by changes of the properties of critical clusters are incorporated via the generalized Gibbs’ approach. It is demonstrated that (iv) a similar formulation of the nucleation theorem, Equation (35), as obtained via the assumptions employed in CNT, Equation (9), also holds true in such cases. However, (v) specific forms of the nucleation theorem, describing the change of the work of critical cluster formation with respect to variations of temperature, pressure, and/or composition, will have a considerably more complex shape. Similarly to the specification of the parameters of critical clusters in crystal nucleation, they have to be appropriately accounted for in order to arrive at a correct method of determination of critical cluster properties via the different forms of the nucleation theorem.
